# Co-ingestion of whole almonds and almond oil with carbohydrate suppresses postprandial glycaemia in mice in an insulin-dependent and insulin-independent manner

**DOI:** 10.1017/jns.2019.22

**Published:** 2019-07-31

**Authors:** Kazuko Kato, Phuong H. T. Vo, Takashi Furuyashiki, Hiroshi Kamasaka, Takashi Kuriki

**Affiliations:** Institute of Health Sciences, Ezaki Glico Co., Ltd., Nishiyodogawa-ku, Osaka 555-8502, Japan

**Keywords:** Almond fractions, Almond skins, Almond oil, Postprandial glycaemia, Postprandial insulinaemia, GLP-1, glucagon-like peptide 1, PGL, plasma glucose level

## Abstract

Co-ingestion of almonds with carbohydrate prevents excessive increase in plasma glucose level (PGL), but information about the functional fraction is limited. Identifying the functional fraction is necessary to use almonds more efficiently in terms of controlling postprandial glycaemia after a high-carbohydrate meal. In the present study, we evaluated the effects of almond skin, oil, water-soluble fraction and water-insoluble fraction on both postprandial glycaemia and insulinaemia. The effect of almond skin was tested by comparing the effect of whole almonds with the effect of skinless almonds. Male ICR mice were administered dextrin and 4 g/kg body weight test samples. After the administration, 2-h postprandial changes in glycaemia and insulinaemia were measured. Oil was the only fraction being able to blunt postprandial glycaemia. Interestingly, when co-ingesting with dextrin, almond oil did not change the insulin level compared with the control but whole almonds or skinless almonds triggered a 4-fold increase in insulin level. The co-ingestion of whole almonds or skinless almonds similarly suppressed the PGL at 15 and 30 min (*P* < 0·05), which means almond skin has no effect on postprandial glycaemia. Neither soluble nor insoluble fractions lead to any significant changes in postprandial glycaemia and insulinaemia. In conclusion, oil is the main functional component accounting for the glycaemia-lowering effect without altering insulin level.

Glucose homeostasis is one of the important factors for our health. High postprandial glycaemia can lead to an increase in fat storage, lethargy and food cravings, which is linked to higher risks of obesity, insulin resistance and diabetes^(^^1^^,^^2^^)^. High postprandial glycaemia is reported as a risk factor for cerebrovascular disease in patients with type 2 diabetes^(^^3^^)^.

The health benefits of almonds are well known. Some studies have shown that the incorporation of almonds into high-carbohydrate meals can decrease postprandial glycaemia in a dose-dependent manner^(^^4^^,^^5^^)^. This effect could be a favourable method for preventing postprandial glycaemic excursion. However, the dietary components of almonds that account for the observed effect are still unclear. Some components of almonds have shown their own health benefits. First, phenolic compounds, which are abundant in almond skins, blunt plasma glucose level (PGL) by decreasing the amylase activity, thus retarding carbohydrate absorption^(^^4^^,^^6^^)^. Second, the dietary fibres in almonds increase the viscosity of the intestinal contents and hinder carbohydrate absorption^(^^7^^)^. Third, the high contents of fats and proteins in almonds have a glycaemia-lowering effect by decreasing gastric emptying rate^(^^8^^)^. Tsujita *et al*.^(^^4^^)^ reported the effect of almond skin polyphenols on inhibiting α-amylase activity. However, a purified form of polyphenols was used in the study and the concentration was higher than that of natural polyphenols in almond skins. Thus far, there is no information about the effect of specific almond components in their natural form as well as their effective concentrations. Furthermore, the effect of almonds on glycaemia is known to be influenced by a number of factors, including the timing of almond intake. The effect of co-ingestion of almonds with carbohydrate has been clearly described in a few studies before^(^^6^^,^^9^^,^^10^^)^. Besides, Crouch & Slater^(^^11^^)^ found that 30 min after almond preloading, the average 1-h PGL decreased approximately 19·4 % in a glucose tolerance test. Nevertheless, whether preload or co-ingestion of almonds with carbohydrate is more effective for decreasing postprandial glycaemia is still unknown. Therefore, in the present study, we conducted a preliminary experiment to determine the best time to feed the mice. Then, we investigated the effects of the almond skins, oil, water-soluble and water-insoluble fractions on postprandial PGL to determine the most effective component.

## Materials and methods

### Almonds and almond fractions

**Fig. 1.**
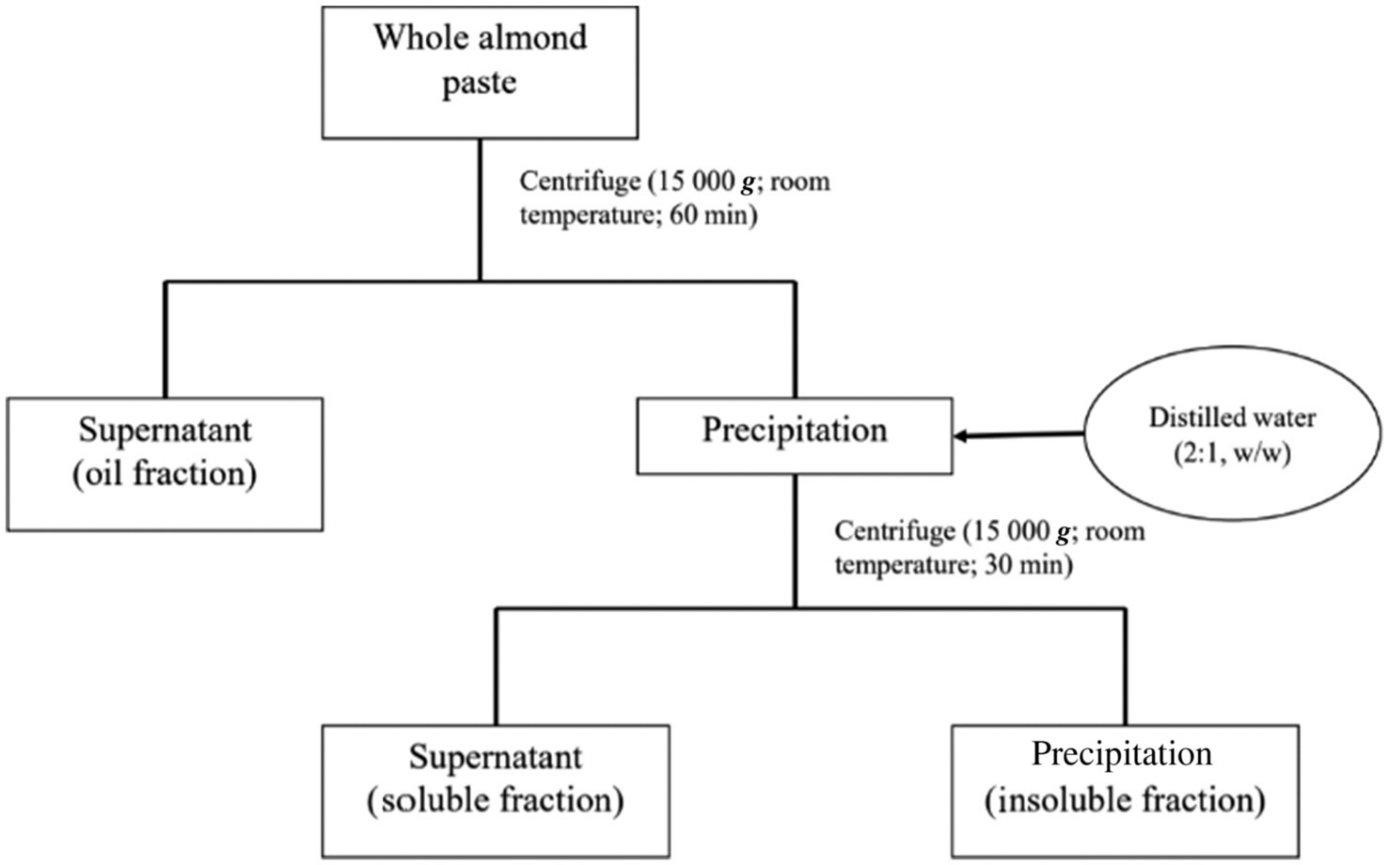
Diagram for the preparation of oil, water-insoluble and water-soluble fractions.

Whole roasted almonds and skinless almonds in paste forms were purchased from Sagami Industry Co., Ltd. The main nutritional composition of 100 g whole almond paste was 21·2 g protein, 57·6 g fat and 16·1 g carbohydrate (6·6 g sugar and 9·5 g dietary fibres). The whole almond paste was centrifuged to harvest three fractions: (a) oil fraction, (b) water-insoluble fraction (insoluble fraction) and (c) water-soluble fraction (soluble fraction) (Fig. 1). The almond dose was 4 g/kg body weight. To facilitate oral administration in mice, the almond paste, soluble fraction and insoluble fraction were diluted to 30, 34 and 29 % (w/w) aqueous solutions, respectively. These prepared samples, derived of almonds, were applied for mice as experimental diet. The nutritional compositions of oral administration solutions are presented in Table 1. The administered dose of oil was 2·26 g/kg body weight.

**Table 1.** Nutrient composition of the oral administration solutions*

Nutrients (g/100 g)	Whole almond paste	Soluble fraction	Insoluble fraction
Water	70·57	95·80	86·10
Protein	6·36	2·20	3·30
Fat	17·28	0·60	6·70
Ash	0·96	0·20	0·60
Carbohydrate	4·83	1·20	3·30
Sugar	1·98	1·10	0·80
Dietary fibre	2·85	0·10	2·50
Energy kcal kJ	196·7 823·0	19 79	82 343

* The data were analysed by Japan Food Research Laboratories (Osaka, Japan).

### Other materials

Following the report that co-ingestion of indigestible dextrin with carbohydrate significantly decreased PGL^(^^12^^)^, 3 % indigestible dextrin solution (Matsutani Chemical Industry Co., Ltd.) was used as a positive control in the preliminary experiment. We used water as the negative control and 10 % dextrin solution (Glico Nutrition Co., Ltd.) as the carbohydrate source. The administered dose of indigestible dextrin was 0·4 g/kg body weight and dextrin was 1 g/kg body weight.

### Animals

ICR mice, aged 6 weeks (male, *n* 30–32, weighing 40–55 g; Japan SLC Inc.) were housed at five or six mice per cage and maintained under standard conditions (23 ± 1°C, 50 ± 10 % humidity, 12-h light–dark cycle). Food and water were provided *ad libitum*, except when fasting was needed. The acclimatisation time was 1 week. All animal experiments were conducted in compliance with protocols no. 2015-0017, 2015-0021 and 2015-0024, reviewed by the Institutional Animal Care and Use Committee of Ezaki Glico Co., Ltd. and Guidelines for Proper Conduct of Animal Experiments (Science Council of Japan) of July 2015.

### Preliminary experiment: the effect of almond-intake timings

As a preliminary experiment, we determined the suitable intake timing of almonds by comparing the effects of 30-min almond preload and almond co-ingestion. The better almond-intake timing was going to be used in the main experiments.

Mice, aged 7 weeks, were fasted for 18 h and randomly allocated to three groups: whole-almond group (*n* 11), indigestible-dextrin group (*n* 11), and water group (*n* 10). The almond preloading test was conducted first. Then, the almond co-ingesting test was conducted 1 week later.

**Fig. 2.**
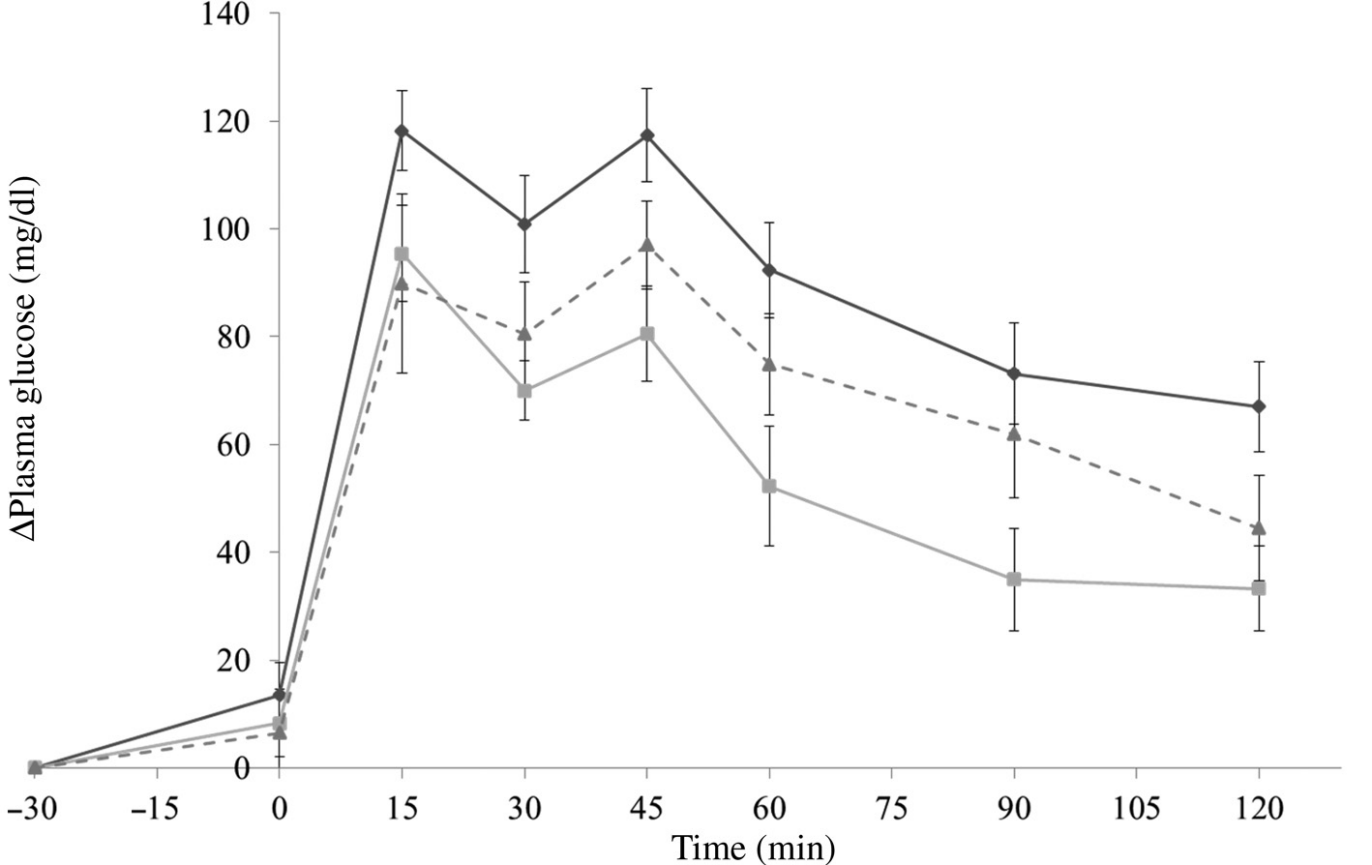
Δ Plasma glucose levels in mice after 30-min preloading with almond (–♦–), indigestible dextrin (–■–) or water (–▲--). Values are means, with their standard errors represented by vertical bars. Whole-almond group, *n* 10–11; indigestible-dextrin group, *n* 9–10; water group, *n* 9–10. To convert plasma glucose in mg/dl to mmol/l, multiply by 0·0555.

**Fig. 3.**
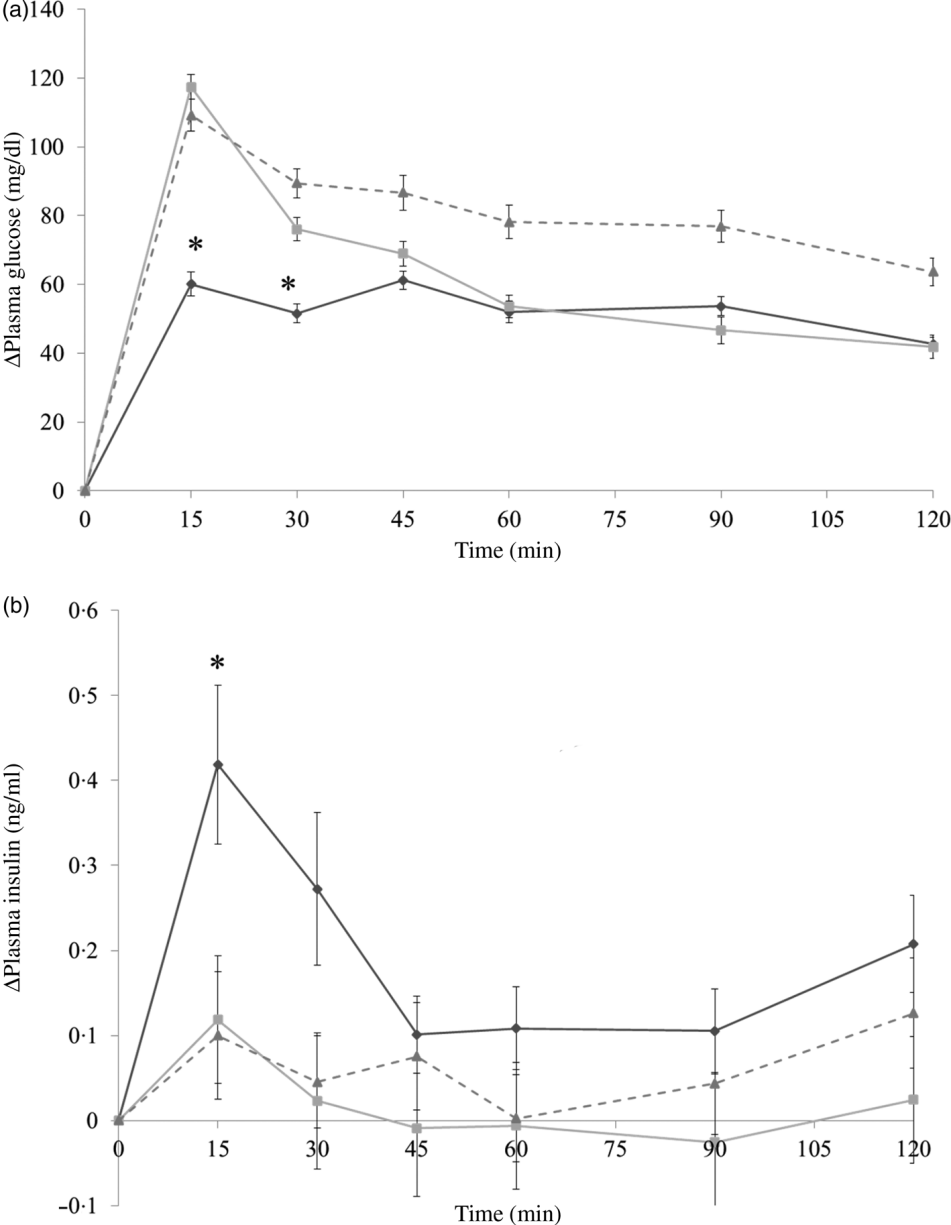
Δ Plasma glucose levels (a) and Δ plasma insulin levels (b) in mice after mice had been administered with dextrin and almond (–♦–), dextrin and indigestible dextrin (–■–) or dextrin and water (–▲--). Values are means, with their standard errors represented by vertical bars. (a) Almond group, *n* 9; indigestible dextrin group, *n* 9–10; water group, *n* 9. (b) Almond group, *n* 10; indigestible dextrin group, *n* 9–10; water group, *n* 10. * Significant difference between the almond and water groups (*P* < 0·05; Dunnett's test). To convert plasma glucose in mg/dl to mmol/l, multiply by 0·0555.

In the preloading test, each group was administered with almonds (4 g/kg body weight) with diluted forms, indigestible dextrin (0·4 g/kg body weight) as a positive control, or the same volume of deionised water via oral administration. At 30 min later, the mice were administered with dextrin solution (1 g/kg body weight). In the co-ingesting test, almonds (4 g/kg body weight) with diluted forms, indigestible dextrin (0·4 g/kg body weight), or deionised water were administered with dextrin solution (1 g/kg body weight) simultaneously. The time point when dextrin solution was administered was set as 0 min. About 800 µl of blood samples were collected from the tail tip at −30, 0, 15, 30, 45, 60, 90 and 120 min and were centrifuged at 5000 ***g*** for 5 min to collect the blood plasma. Blood plasma was stored at −80°C until further analysis. Although the details should be mentioned in the following results section, the co-ingestion of almonds showed the better effect at suppressing PGL (Fig. 2 and Fig. 3). Hence, we conducted the main experiments by administering almonds or almond fractions at the same time with dextrin.

### Experiment 1: the effect of almond skin

To evaluate the effect of almond skin without changing its natural form and concentration, we compared the effect of whole almonds with the effect of skinless almonds in experiment 1. After 18 h of fasting, the 7-week-old mice were divided into three groups: whole-almond group (*n* 13), skinless-almond group (*n* 13) and water group (*n* 14). A certain amount (4 g/kg body weight) of whole almonds or skinless almonds, with diluted forms, was administered to the mice following dextrin solution (1 g/kg body weight). Blood samples from the tail tip were collected at 0, 15, 30, 45, 60, 90 and 120 min. Plasma samples were collected and handled in the same way as in the preliminary test.

### Experiment 2: the effect of oil, water-soluble and water-insoluble fractions

The mice in experiment 1 (*n* 30) were re-used in experiment 2 at a 1-week interval. After the 18-h overnight fast, 8-week-old mice were divided into four groups: oil fraction group (*n* 8), soluble fraction group (*n* 8), insoluble fraction group (*n* 7) and water group (*n* 7). Oil fraction, soluble fraction, insoluble fraction, or deionised water, with diluted forms, was administered to the mice accordingly. The mice were then given 1 g/kg body weight dextrin solution. After administration, blood samples were collected and handled in the same way as in the preliminary test.

### Measurement of plasma glucose and insulin

Plasma glucose and insulin levels were measured, following the standard protocol, using the glucose C-II test Wako kit (Wako Pure Chemical Industries, Ltd.) and Mouse Insulin ELISA kit (Morinaga Institute of Biological Science, Inc.), respectively. Changes in plasma glucose and insulin levels were calculated by subtracting the fasting value and shown as incremental (Δ) changes in each figure.

### Statistical analyses

All values are means with their standard errors. The changes in postprandial glycaemia and insulinaemia were compared at different time points between the tested groups and the water group by means of Dunnett's test at *P* < 0·05. Statistical analyses were performed using JMP software (version 14.2.0).

## Results and discussion

**Fig. 4.**
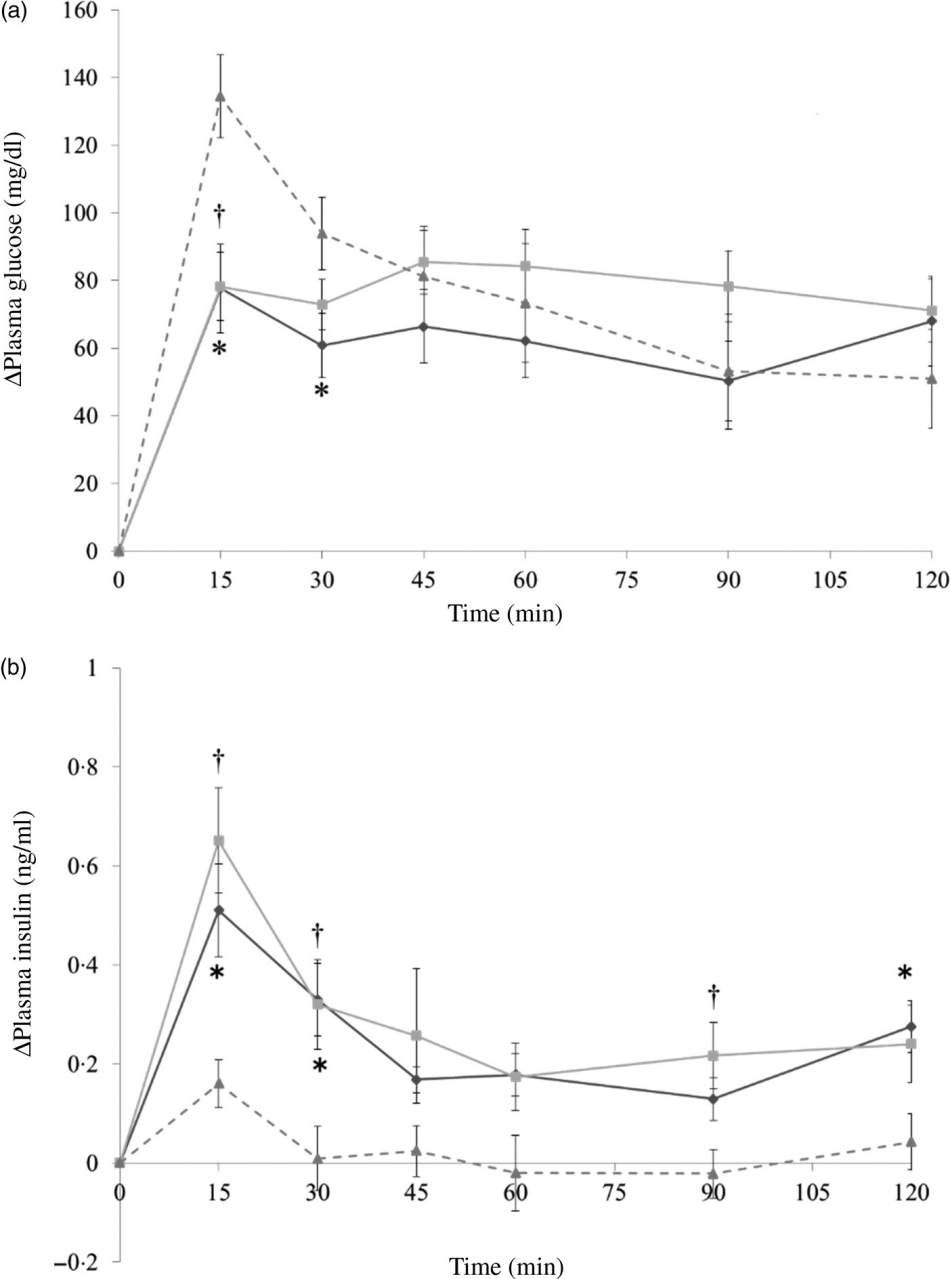
Δ Plasma glucose levels (a) and Δ plasma insulin levels (b) in mice after mice had been administered with dextrin and whole almond (–♦–), dextrin and skinless almond (–■–) or dextrin and water (–▲--). Values are means, with their standard errors represented by vertical bars. (a) Whole almond group, *n* 12–13; skinless almond group, *n* 12–13; water group, *n* 12–14. (b) Whole almond group, *n* 10–11; skinless almond group, *n* 8–9; water group, *n* 8–9. * Significant difference between the whole almond and water groups (*P* < 0·05; Dunnett's test). † Significant difference between the skinless almond and water groups (*P* < 0·05; Dunnett's test). To convert plasma glucose in mg/dl to mmol/l, multiply by 0·0555.

The administration of both whole and skinless almonds led to the same result of decreasing glycaemic response (Fig. 4(a)). Whole almonds significantly decreased PGL at 15 and 30 min. For example, at 15 min, the PGL of the whole-almond group was 77·6 mg/dl (4·3 mmol/l), and that of the water group was 134·4 mg/dl (7·5 mmol/l). Similarly, the value of PGL in the skinless-almond group was significantly lower than that in the water group at 15 min. The similarity of changing trend in glycaemic response between whole almond and skinless almonds implies that almonds skin is not the component responsible for the glycaemia-lowering effect.

**Fig. 5.**
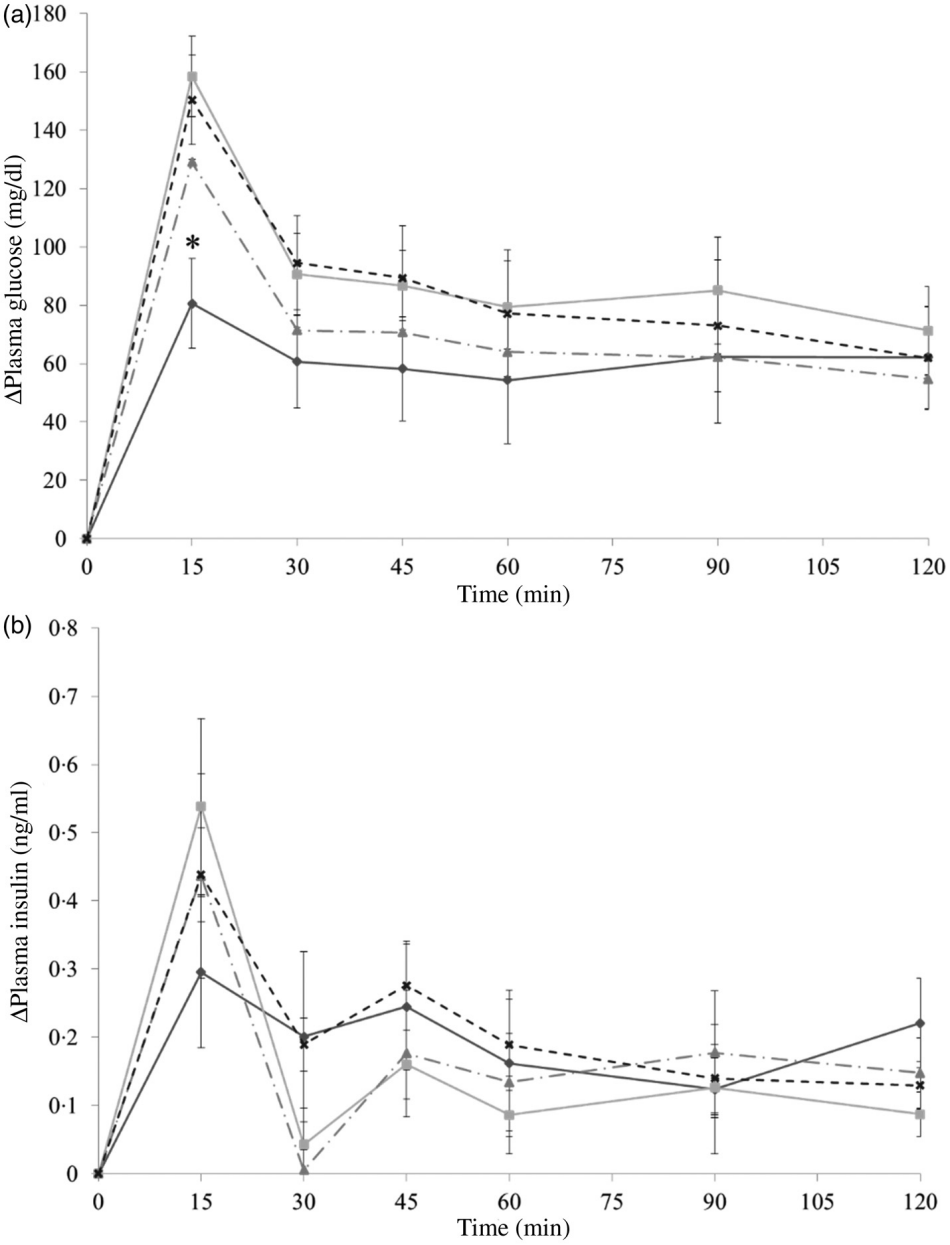
Δ Plasma glucose levels (a) and Δ plasma insulin levels (b) in mice after mice had been administered with dextrin and oil fraction (–♦–), dextrin and soluble fraction (–■–), dextrin and insoluble fraction (–▲--) or dextrin and water (–x--). Values are means, with their standard errors represented by vertical bars. (a) Oil group, *n* 8; soluble group, *n* 8; insoluble group, *n* 7; water group, *n* 8. (b) Oil group, *n* 8; soluble group, *n* 8; insoluble group, *n* 7; water group, *n* 8. * Significant difference in plasma glucose levels between the almond oil and water groups (*P* < 0·05; Dunnett's test). To convert plasma glucose in mg/dl to mmol/l, multiply by 0·0555.

The results in experiment 2 showed that almond oil was able to reduce postprandial glycaemia when co-ingesting with dextrin. As shown in Fig. 5(a), in comparison with the PGL of the water group, the only significant difference from the control was observed in the oil-fraction group. At 15 min, the PGL of the water group was 150·3 (se 15·4) mg/dl (8·3 (se 0·9) mmol/l), which is approximately two times higher than that of the oil fraction group (80·6 (se 18·06) mg/dl (4·5 (se 1·0) mmol/l)).

Decreasing postprandial glycaemia by the incorporation of fat into the meal has been reported in previous studies^(^^8^^,^^13^^,^^14^^)^. Fat decreases the gastric emptying rate, which is related strictly to the glucose level response^(^^15^^)^. The slow gastric emptying rate could be the result of (1) hormone responses to the presence of fat in the small intestine and/or (2) high energy content of fat. It is widely accepted that the presence of fatty acids in the ileum increases the secretion of gastrointestinal hormones. When 50 g fat was co-ingested with 50 g carbohydrate, Collier & O'Dea^(^^8^^)^ observed an increase in the level of gastric inhibitory polypeptide (GIP), a hormone that plays a role in decreasing gastric emptying rate. Gunnarsson *et al*.^(^^16^^)^ also reported that MUFA increased the level of intact glucagon-like peptide 1 (GLP-1), an ileal brake hormone associated with slowing the gastric emptying rate. The high energy content of fat could also induce a slower gastric emptying rate. The relationship between meal energy content and gastric emptying rate has been demonstrated in some studies before^(^^17^^,^^18^^)^. Nevertheless, the main pathway that is responsible for this effect still remains unknown.

In the present study, the hormone response could hardly be the case. The result of insulin level in experiment 2 (Fig. 5(b)) showed that almond oil suppressed postprandial glycaemia without changing the insulin level, whereas insulin is associated with incretin hormones, such as GLP-1. This indicates that almond oil in the carbohydrate meal could slow gastric emptying because of its high energy content and viscosity.

As suggested by the results of the study by Gatti *et al*.^(^^19^^)^, different types and sources of fat lead to different effects on glycaemia. It raises the question whether almond oil has stronger postprandial glycaemia suppression effect over other nut oils. In a separate experiment using the same study design, we compared the effect of almond oil with the effect of peanut oil, walnut oil and olive oil (data not shown). The postprandial glycaemia in response to almond oil, walnut oil and olive oil showed the same trend. The PGL values were suppressed at 15 min after administration. Nevertheless, peanut oil did not suppress the PGL during the postprandial period. Although almond oil, walnut oil, olive oil and peanut oil all mostly consist of unsaturated fatty acids, the type of unsaturated fatty acids is different. The composition of fatty acids is possibly the main factor that causes the observed results.

It is noticeable that whole almonds suppressed plasma glucose at 15 and 30 min (Figs. 3(a) and 4(a)) whereas the oil fraction only suppressed plasma glucose at 15 min (Fig. 5(a)). In experiment 1, at 15 min, the PGL of the whole-almond group was 60·1 (se 3·43) mg/dl (3·3 (se 0·19) mmol/l), whereas that of the indigestible-dextrin and water groups were 117·4 (se 3·53) and 109·2 (se 4·72) mg/dl (6·5 (se 0·20) and 6·1 (se 0·26) mmol/l), respectively. At 30 min, the PGL in the whole-almond group was slightly decreased to 51·5 (se 2·71) mg/dl (2·9 (se 0·15) mmol/l) and still maintained a significantly lower PGL than the water group (Fig. 3(a)). On the other hand, the result of experiment 2 showed that even the PGL of the oil fraction group at 15 min was significantly lower than that of the water fraction, the difference disappeared soon and the effect of oil could not been seen any more (Fig. 5(a)). This implies that whole almonds are more effective than almond oil in terms of decreasing postprandial glycaemia. The difference in the glycaemia response between whole almonds and almond oil could be explained by the contribution of almond protein.

Similar to almond oil, almond protein could probably decrease postprandial glycaemia by slowing the gastric emptying rate. Protein is one of the proven macronutrients that induces the secretion of incretin hormones such as gastric inhibitory polypeptide (GIP) and GLP-1. Then, this increase in incretin hormones eventually decreases the gastric emptying rate^(^^15^^,^^20^^)^. When administrating glucose alone and glucose with whey protein, the gastric emptying rate was decreased from 8·4 to 4·0 mmol/l, together with the increase in intact GLP-1 level^(^^16^^)^.

In addition to slowing the gastric emptying rate, almond protein could also be capable of triggering insulin secretion. At 15 min after administration, there was a sharp increase in insulin level of the almond group (Fig. 3(b) and Fig. 4(b)). The co-ingestion of protein could induce the release of incretin hormones like insulin. Gunnarsson *et al*.^(^^16^^)^ demonstrated that the insulin responses to glucose could increase 1·5 times in the presence of additional whey protein. Further analysis focused on almond protein and almond amino acids should be taken into consideration to clarify this possibility.

The dispersal of protein into two fractions could explain the reason why there was no significant increase in the insulin level in experiment 2 (Fig. 5(b)). The almond protein level in these fractions probably fell under the insulinotropic threshold. When a significant increase in the insulin level was observed after protein and glucose administration, the ratio of protein to carbohydrate was generally higher than 0·5. For example, this ratio was 1 (75 mg whey protein/75 mg d-glucose) and 0·728 (18·2 g protein/25 g carbohydrate) in the experiments by Gunnarsson *et al*.^(^^16^^)^ and Nilsson *et al*.^(^^20^^)^, respectively. In the present study, the ratio of almond protein to dextrin was 0·846 in the whole almond group (preliminary experiment and experiment 1); however, it decreased to 0·293 in the soluble-fraction group and 0·439 in the insoluble-fraction group (experiment 2).

Overall, the changing pattern of postprandial insulinaemia in whole almonds and oil-fraction group suggested that almonds suppress postprandial glycaemia in both an insulin-dependent manner and an insulin-independent manner. Triggering insulin secretion is probably due to almond protein. Slowing gastric emptying rate could be another mechanism and is attributable to both oil and protein constituents, as discussed above. Nevertheless, it is difficult to conclude which mechanism plays the more important role. Research in which both gastric emptying rate and insulin level are measured is required to clarify the main mechanism.

### Conclusions

Although oil alone could not explain for the complete glycaemia-lowering effect of almonds, it is the major beneficial fraction. When considering almonds as a whole, triggering insulin secretion is one of the mechanisms and almond protein is predicted as the cause of insulin elevation. By comparing insulin changes in response to oil and whole almonds, it is suggested that oil's glycaemia-lowering effect could relate to the gastric emptying rate.
